# Biocatalytic Synthesis of Antiviral Nucleosides, Cyclic Dinucleotides, and Oligonucleotide Therapies

**DOI:** 10.1021/jacsau.2c00481

**Published:** 2022-11-30

**Authors:** Kyle J. D. Van Giesen, Matthew J. Thompson, Qinglong Meng, Sarah L. Lovelock

**Affiliations:** Manchester Institute of Biotechnology, School of Chemistry, University of Manchester, 131 Princess Street, Manchester M1 7DN, U.K.

**Keywords:** biocatalysis, nucleosides, cyclic dinucleotides, oligonucleotides, nucleic acid therapeutics

## Abstract

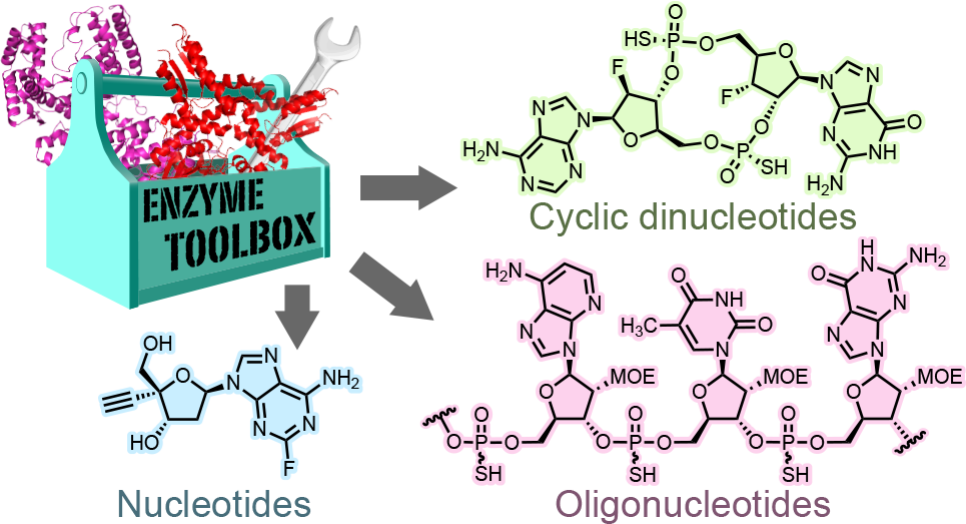

Nucleosides, nucleotides, and oligonucleotides modulate diverse cellular processes ranging from protein production to cell signaling. It is therefore unsurprising that synthetic analogues of nucleosides and their derivatives have emerged as a versatile class of drug molecules for the treatment of a wide range of disease areas. Despite their great therapeutic potential, the dense arrangements of functional groups and stereogenic centers present in nucleic acid analogues pose a considerable synthetic challenge, especially in the context of large-scale manufacturing. Commonly employed synthetic methods rely on extensive protecting group manipulations, which compromise step-economy and result in high process mass intensities. Biocatalytic approaches have the potential to address these limitations, enabling the development of more streamlined, selective, and sustainable synthetic routes. Here we review recent achievements in the biocatalytic manufacturing of nucleosides and cyclic dinucleotides along with progress in developing enzymatic strategies to produce oligonucleotide therapies. We also highlight opportunities for innovations that are needed to facilitate widespread adoption of these biocatalytic methods across the pharmaceutical industry.

## Introduction

**Figure 1:**
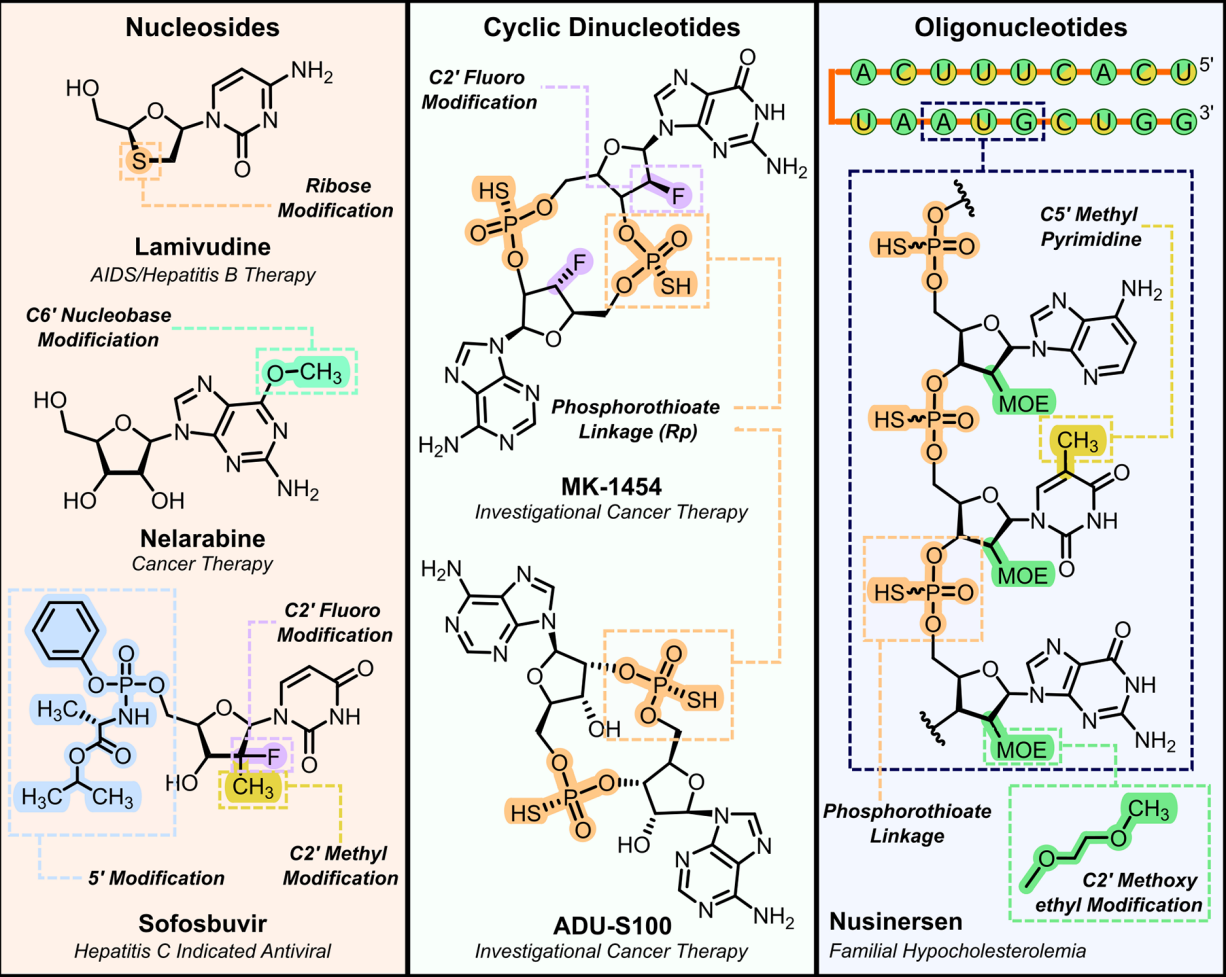
Structures of nucleoside, cyclic dinucleotide, and oligonucleotide therapeutics. Common chemical modifications used to improve pharmacodynamic and pharmacokinetic properties are highlighted.

Nucleotides are a central building block of life. They make up our DNA and RNA that in turn encode the proteins required to control biochemical processes and are also used as enzyme cofactors and as a source of energy. Synthetic analogues of nucleosides, nucleotides, and oligonucleotides, herein referred to as nucleic acids, have been developed as therapeutics to target diverse processes including DNA replication, transcription, translation, and cell signaling (Figure 1).^1−9^ Nucleosides, comprising a ribose sugar and nucleobase, are well established as anticancer and antiviral therapies.^1−3^ For example, lamivudine and nelarabine are nucleoside prodrugs that are converted to the corresponding nucleotide triphosphates (NTPs) *in vivo* and incorporated into DNA by polymerases. These noncanonical nucleotides inhibit further chain extension, thus preventing DNA replication and inhibiting cell or viral proliferation. Cyclic dinucleotides (CDNs) are secondary signaling molecules which control many processes in both prokaryotes and eukaryotes and are comprised of two ribonucleic acid monophosphates linked via 3′–5′ and 2′–5′ phosphodiester bonds.^4−6^ CDNs activate the stimulator of interferon genes (STING) protein to elicit an immune response and are currently being evaluated as potential cancer treatments.^10−12^ While nucleoside and nucleotide analogues commonly target proteins to elicit their therapeutic effects, oligonucleotides target mRNA to modulate the production of disease related proteins.^7−9^ Therapeutic oligonucleotides, which are typically 20 nucleobases in length and can be single stranded (antisense oligonucleotides) or double stranded (siRNAs), have attracted much attention in recent years. Although initially developed to treat rare diseases, therapies for more common disorders have started to emerge, as highlighted by the recent approval of inclisiran for the treatment of atherosclerotic cardiovascular disease.^13,14^

Nucleic acid therapeutics contain chemical modifications to the ribose sugar, nucleobase and/or phosphate backbone, which confer favorable selectivity, binding affinity, metabolic stability, and toxicity profiles.^2,6,15−17^ Common modifications include 2′-ribose substitutions, designed to enhance metabolic stability and promote a stable RNA-like C3′-endo sugar conformation for improved binding affinity. CDNs and oligonucleotides also frequently contain modified phosphate linkages such as phosphorothioates, where the phosphate nonbridging oxygen is substituted by a sulfur.^18−20^ These backbone modifications lead to improved resistance to nuclease degradation and increased hydrophobicity, which improves cellular uptake. Despite recent progress in the chemical synthesis of nucleic acid therapeutics,^21−27^ developing economical and sustainable routes remains a major challenge.^4,6,14,28−30^ Nucleic acids are structurally complex, contain multiple stereogenic centers, and have poor solubility in organic solvents. Synthetic routes typically require extensive protecting group chemistry due to the presence of multiple functional groups, which leads to poor step- and atom-economy. Selectively introducing chemical modifications to the ribose ring can often be particularly challenging, and modifying the phosphate backbone introduces new stereochemical elements which further complicate synthesis. In the case of therapeutic oligonucleotides, scalable manufacturing remains a major hurdle, with current methods relying on solid-phase phosphoramidite chemistry that uses four steps per base extension, uses prohibitively large volumes of acetonitrile (1000 kg per kg of oligonucleotide), and is restricted to <10 kg batches.^30−32^ In light of these challenges, biocatalysis has emerged as an attractive and sustainable alternative to traditional chemical methods. Biocatalysts operate under aqueous conditions that are compatible with the synthesis of polar molecules and offer unrivalled levels of stereo- and regiocontrol, thereby alleviating the need for protecting groups.^33−37^ Given the structural similarity to biomolecules found in nature, there is an abundance of enzymes and biosynthetic pathways that can serve as inspiration for the development of new biocatalytic approaches to nucleic acid analogues. Here, we highlight recent examples of nucleoside, CDN, and oligonucleotide synthesis using biocatalysis that serve to illustrate the benefits of applying enzymatic methods for the preparation of this important class of therapeutics.

## Nucleosides

**Figure 2:**
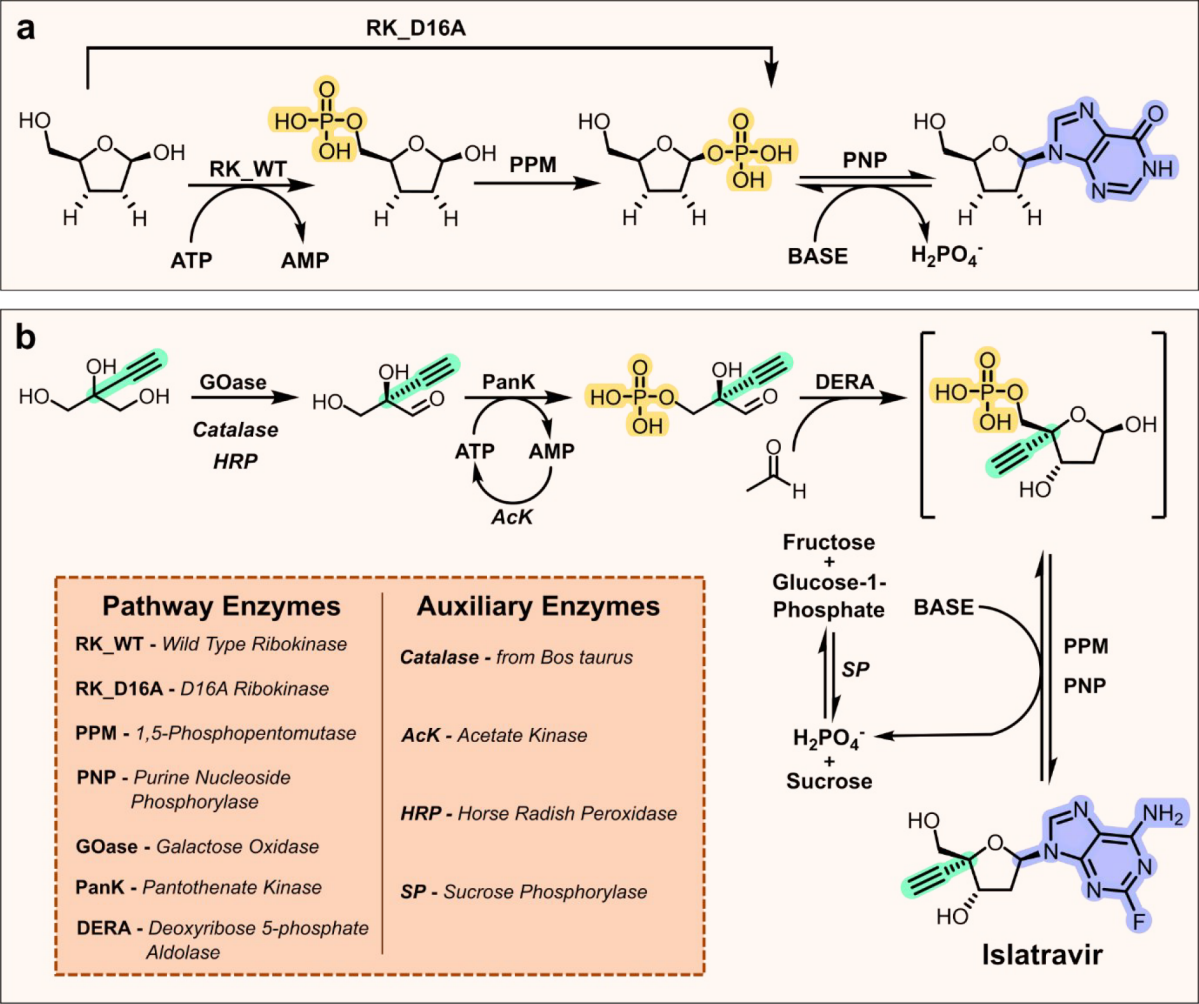
Biocatalytic approaches to nucleoside analogues. (a) Enzyme cascade for the synthesis of didanosine involving a ribokinase (RK), phosphopentomutase (PPM), and purine nucleoside phosphorylase (PNP). The RK variant D16A has mechanistic promiscuity and can catalyze direct 1′-ribose phosphorylation, avoiding the requirement for PPMs. (b) Enzyme cascade for the synthesis of Islatravir. The first step involves a galactose oxidase (GOase) catalyzed desymmetrization using auxiliary enzymes catalase and HRP. In the second step, pantothenate kinase (PanK) catalyzes substrate phosphorylation using ATP, which is recycled by acetate kinase (AcK) and acetylphosphate. The final step involves deoxyribose 5-phosphate aldolase (DERA), PPM and PNP, and a sucrose phosphorylase (SP) auxiliary enzyme to drive the equilibrium.

Enzymes from nucleoside metabolism and salvage pathways have inspired the development of a general enzymatic cascade that has been used for the synthesis of several nucleoside therapeutics.^38−40^ First, ribokinases (RK) catalyze the 5′-phosphorylation of ribose using ATP as a cosubstrate. The 5′-phosphate group is then transferred to the C1′-OH using a phosphopentomutase (PPM). Finally, the nucleobase is installed in a stereocontrolled fashion at the C1-position using a nucleoside phosphorylase (PNP) (Figure 2a). Stoichiometric ATP used by RKs in step 1 is inhibitory to PPM activity. To address this limitation and to improve process efficiency, catalytic ATP and cofactor recycling enzymes such as acetate kinases (AcK) and pyruvate kinases (PK) are commonly employed. Performing steps in tandem avoids the need to isolate intermediates, improves step economy, and can also improve the efficiency of steps with unfavorable equilibrium constants, as intermediates generated can be reacted in downstream transformations.^41,42^ PNPs have been well characterized and are known to tolerate a range of 4′- and 2′-modified ribose sugars as well as modified nucleobases, making them attractive biocatalysts for manufacturing nucleoside therapeutics.^43−46^ The RK, PPM, and PNP cascade was applied to the synthesis of 2′,3′-dideoxyinosine (didanosine), an off-patent HIV therapy (Figure 2a).^38,46^ Here the pathway enzymes were engineered for improved selectivity toward the target dideoxy-modified substrate to enable nucleoside synthesis *in vivo*. Interestingly, a single D16A active site mutation in *E. coli* RK gave rise to 1′-phosphorylation activity, meaning that didanosine could be accessed in a simple two step RK and PNP process avoiding the need for PPMs.

The general enzyme cascade outlined above has also been extended for the synthesis of islatravir, an investigational HIV reverse transcriptase translocation inhibitor.^39,47^ Islatravir contains a C4′-ethynyl substitution on the ribose ring, and as such, additional steps were required to synthesize the modified sugar (Figure 2b). First, an engineered galactose oxidase (GOase) was used to desymmetrize 2-ethynylglycerol. Here catalase was used to sequester the hydrogen peroxide byproduct and horse radish peroxidase (HRP) was used to maintain the correct oxidation state of the GOase copper cofactor. The 2-ethynyl glyceraldehyde product was then phosphorylated using pantothenate kinase (PanK) and catalytic ATP, which was regenerated using AcK and an acetylphosphate donor. To complete the synthesis of the ribose ring, deoxyribose 5-phosphate aldolase (DERA), catalyzed the stereoselective aldol reaction between 2-ethynyl glyceraldehyde 3-phosphate and acetaldehyde. Finally, PPMs and PNPs were used to install the 2-fluoroadenine nucleobase. PNP-catalyzed reactions are reversible,^48^ so to drive the reaction to completion the phosphate byproduct was sequestered using sucrose phosphorylase (SP). All five on-pathway enzymes were engineered for improved efficiency toward the 4′-ethynyl modification. Optimization of the GOase was particularly impressive, with 34 mutations installed into the starting GOase F2 scaffold over 12 rounds of directed evolution,^49^ resulting in 11-fold improvement in activity, reduced product inhibition, and inversion of stereochemistry to provide access to the desired (*R*)-2-ethynyl glyceraldehyde product with high levels of stereocontrol (90:10 *R*:*S* selectivity). The final synthesis involved nine enzymes in total and was performed in three steps to provide islatravir in an impressive 51% isolated yield.

**Figure 3:**
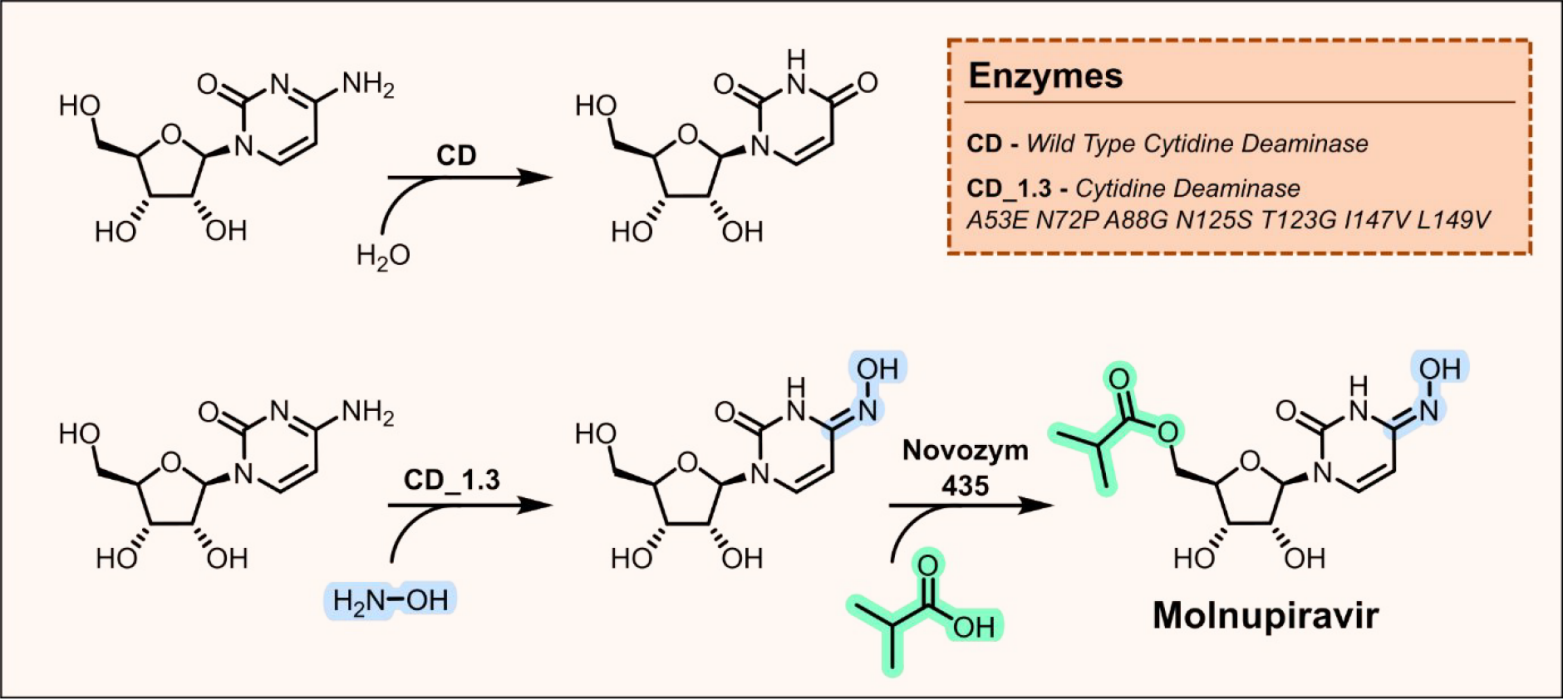
Cytidine deaminase (CD) catalyzed production of a key molnupiravir intermediate. In nature, CD catalyzes the hydrolysis of cytidine to uridine. CD was engineered to optimize its promiscuous hydroxyamination activity and provide direct access to *N*-hydroxycytidine from cytidine. A second lipase catalyzed 5′-acylation step delivers the target molnupiravir.

Molnupiravir is a recently approved oral therapy for the treatment of SARS-CoV2 developed in partnership between Ridgeback Biotherapeutics and Merck & Co.^50^ The original synthesis consisted of 10 steps and was limited by low process productivity and high costs.^51^ In light of the urgent need to tackle the covid-19 pandemic, several groups began to develop more sustainable and cheaper biocatalytic manufacturing routes. One strategy developed by Merck involved a three-step chemoenzymatic approach, starting with a selective 5′-OH acylation of ribose catalyzed by the commercial lipase Novozym 435. Next an enzyme cascade involving an engineered kinase and uridine phosphorylase was used to install a uridine nucleobase. Finally, modification of the nucleobase to install the requisite hydroxylamine motif was achieved using a chemical transformation with NH_2_OH and hexamethyldisilazane, with *in situ* silylation of the 2′- and 3′-hydroxyl groups to aid product isolation.^40^ An alternative two-step biocatalytic approach to molnupiravir was developed by Burke et al., who developed an engineered enzyme for the conversion of cytidine to the key molnupiravir intermediate *N*-hydroxycytidine.^52^ The authors discovered that the zinc-dependent enzyme cytidine deaminase (CD), which naturally catalyzes the hydrolysis of cytidine to uridine, was mechanistically promiscuous and can also promote hydroxyaminolysis of cytidine and uridine to generate *N*-hydroxycytidine (Figure 3). Initial screening with the wild-type enzyme performed using 10% hydroxylamine in bulk water gave a 1:6 ratio of *N*-hydroxycytidine:uridine, with the enzyme catalyzing rapid hydrolysis of cytidine to uridine followed by slower uridine hydroxyaminolysis to generate an equilibrium distribution of products. To boost *N*-hydroxycytidine production, enzyme engineering was performed to enhance cytidine hydroxyamination activity while minimizing undesired cytidine hydrolysis. Following three rounds of directed evolution an improved variant was identified containing seven active-site mutations (CD1.3), which was able to efficiently catalyze direct hydroxyamination of cytidine to provide an 8:1 *N*-hydroxycytidine:uridine mixture using a 10% hydroxylamine solution in water. Upon reaction intensification, it was observed that the *N*-hydroxycytidine product crystallized from the biotransformation mixture *in situ*, offering a simple means of product isolation. Following reaction scale-up and optimization, CD1.3 catalyzed hydroxyamination was performed on a 900 mL scale to afford 137g of the *N*-hydroxycytidine product in >95% purity. Subsequent selective 5′-OH acylation using Novozym 435 completed this concise synthesis of molnupiravir.

## Cyclic Dinucleotides

**Figure 4:**
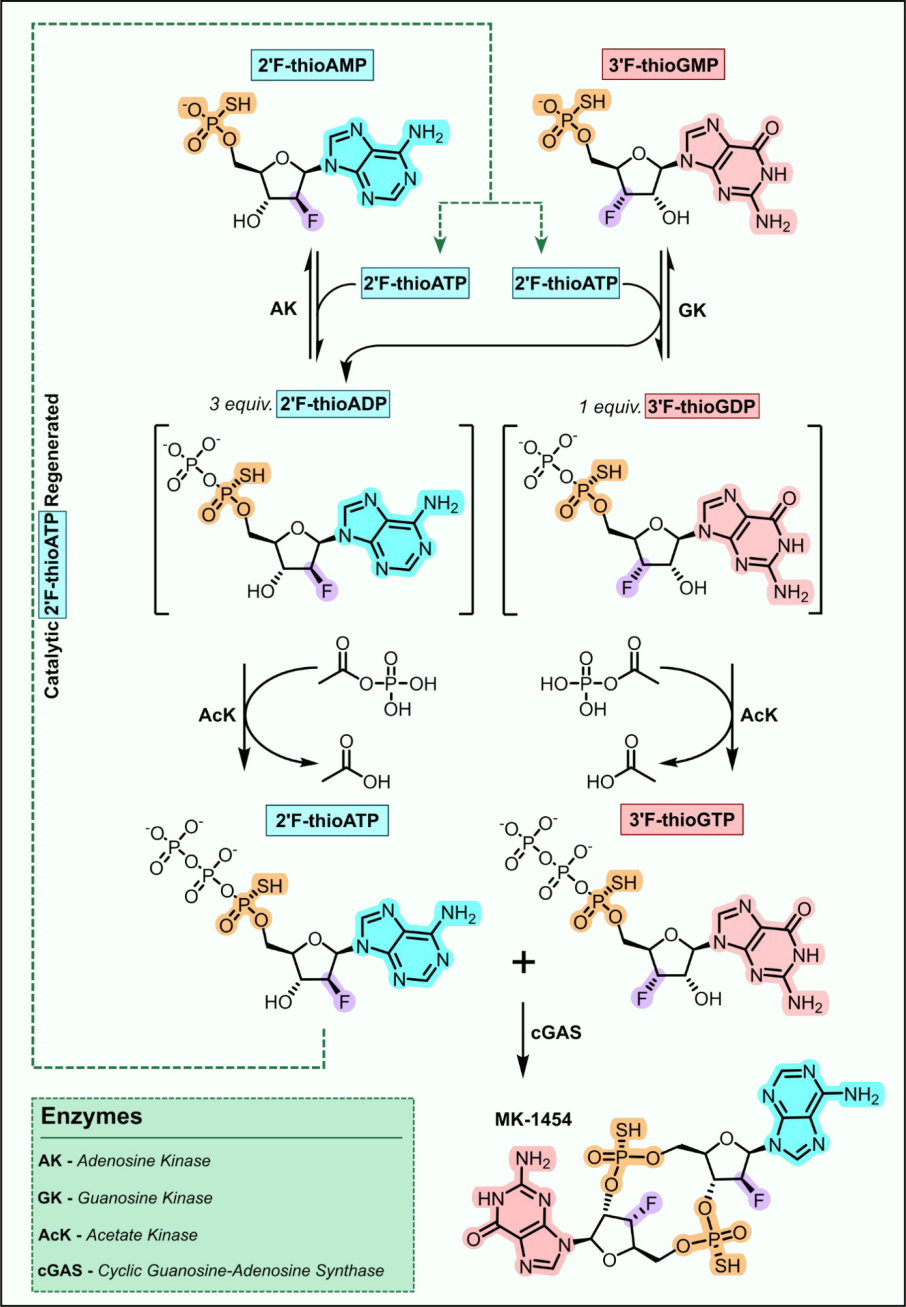
Enzyme cascade for the synthesis of the cyclic dinucleotide MK-1454. Engineered adenylate kinase (AK) and guanylate kinase (GK) were used to convert the 2′-fluoro modified α-thio-monophosphates to 2′F-thio-ADP and 2′F-thio-GDP, respectively, using 2′F-thio-ATP as a phosphate donor. Acetate kinase (AcK) catalyzed the final phosphorylation step using acetylphosphate to provide the α-thiotriphosphates, which were subsequently coupled using an engineered cyclic guanosine-adenosine synthase (cGAS).

Chromatin produced by tumor cells and pathogenic DNA that enters the cytosol are detected by cyclic guanosine monophosphate-adenosine monophosphate synthases (cGASs).^53^ cGAS is activated upon binding to the invading DNA and catalyzes the synthesis of 2′,3′-cyclic guanosine monophosphate-adenosine monophosphate (cGAMP) from ATP and GTP.^54,55^ cGAMP is a secondary signaling molecule that binds to the STING receptor and triggers an immune response.^5,56^ As such, cGAMP analogues are being evaluated as immunotherapy cancer treatments and antiviral agents.^4−6,57^ A prominent example is the cGAMP analogue MK-1454, developed by Merck as an immuno-oncology treatment.^12,58,59^ MK-1454 contains a 2′-fluoro-modified AMP unit, a 3′-fluoro-modified GMP, and two *R*_*p*_ phosphorothioate linkages, designed to improve metabolic stability, bioavailability, and potency. The chemical synthesis of MK-1454 poses significant challenges where two unique nucleotides must be selectively coupled through unsymmetrical 3′–5′ and 2′–5′ phosphorothioate bonds.^60,61^ The original synthesis involved 9 steps and required separation of the desired diastereoisomer by HPLC purification. To improve scalability, step economy, and overall yield, McIntosh et al. set out to develop an *in vitro* biocatalytic cascade exploiting kinases in conjunction with an engineered mammalian cGAS (Figure 4).^62^ The initial results suggested that while cGAS was active toward *S*_*p*_-thio-ATP as a substrate, *S*_*p*_-thio-GTP was not accepted due to an unfavorable interaction between the *S*_*p*_ sulfur atom and the Mg^2+^ cofactor. Exchanging the Mg^2+^ ions for a mixture of Zn^2+^ and Co^2+^ provided access to the desired *R*_*p*_*,R*_*p*_-dithio-cGAMP product via a mechanism which proceeds with inversion of stereochemistry at the P(V) centers. Following 9 rounds of evolution, a variant with sufficient activity toward the required fluoro-modified substrates was identified and used to produce MK-1454 on a 100 mg scale in 90% yield.

Chemical phosphorylation of nucleosides is challenging, so to access scalable quantities of stereopure *S*_*p*_-2’F-thio-ATP and *S*_*p*_-3′F-thio-GTP substrates, engineered kinases were employed to generate the required NTPs from the corresponding nucleoside monothiophosphates.^62−64^*Saccharomyces cerevisiae* adenylate kinase (*Sc*-AdK) and *Branchistoma floridae* guanylate kinase (*Bf*-GK) were used to produce the nucleotide α-thio-diphosphates *S*_*p*_-2’F-thio-ADP and *S*_*p*_-3′F-thio-GDP, respectively, which were subsequently converted to the analogous α-thiotriphosphates using *Thermotoga maritima* acetate kinase (*Tm*-AK). ATP is the canonical phosphate source used by *Sc*-AdK and *Bf*-GK; however, ATP can also react in the cGAS mediated coupling/cyclization step to form undesired side products. To avoid these selectivity issues, an elegant solution was found employing catalytic *S*_*p*_-2’F-thio-ATP as an alternative phosphate donor, which was recycled using *Tm*-AK and acetyl phosphate. Following optimization of reaction conditions, the biocatalytic cascade involving the synthesis of the two NTP substrates and subsequent nucleotide coupling/cyclization reactions were performed in an impressive one-pot reaction to produce MK-1454 in 62% yield.

## Oligonucleotides

Oligonucleotides are the largest (7000–14000 Da) and most structurally complex of the nucleic acid therapeutics and depending on their mode of action contain different structural features.^7−9,65−67^ Antisense oligonucleotides (ASOs) are single-stranded molecules and include gapmers (e.g., mipomersen and inotersen) and steric blockers (e.g., nusinersen). Gapmers inhibit protein production by targeting mRNA and inducing its degradation by RNaseH. They contain a central block of deoxyribose nucleotides required for RNaseH recognition and flanking sequences of 2′-ribose modified nucleotides that provide improved metabolic stability. Gapmers are commonly phosphorothioate modified to improve cellular uptake and increase resistance to nuclease degradation. Steric blocking oligonucleotides hybridize to target mRNA or pre-RNA and block access from the cellular translation machinery without inducing degradation. In this way, steric blockers can modulate splicing, restore protein production, or downregulate gene expression. For example, nusinersen (shown in Figure 1) is a splice-switching oligonucleotide approved for the treatment of spinal muscular atrophy (SMA), a genetic disorder caused by mutations to the SMN1 gene and inefficient production of survival motor neuron (SMN) protein. Nusinersen targets pre-mRNA and redirects splicing to enable the paralogous SMN2 gene to generate functional SMN protein. Steric blockers typically contain uniform modifications along the sequence and commonly include 2′-*O*-methoxyethyl modifications in combination with phosphorothioate linkages (e.g., nusinersen) or a phosphoramidate morpholino backbone (e.g., eteplirsen). Small interfering RNAs (siRNAs, e.g., inclisiran) are a family of double-stranded oligonucleotide therapies that interact with the RNA interference (RNAi) pathway. After binding to the Argonaute 2 protein (AGO2) an RNA-induced silencing complex is formed, which binds to and degrades target mRNA to prevent translation.^68^ siRNAs typically contain multiple types of chemical modifications, most commonly 2′-methoxy and 2′-fluoro, which are dispersed throughout the RNA double stranded sequence. These modifications are needed to enhance metabolic stability and improve binding affinity but must not interfere with recognition by the RNAi system.

Oligonucleotide synthesis currently makes use of phosphoramidite building blocks and relies on iterative rounds of coupling, capping, oxidation/sulfurization, and deprotection to extend sequences immobilized on a solid support.^69^ While this approach provides a flexible means to access modified sequences it is severely limited by sustainability, scalability, and its ability to generate stereochemically defined oligonucleotides.^30−32,70^ Biocatalysis could offer an attractive solution to the oligonucleotide manufacturing challenge, allowing the production of high purity oligonucleotides in a more atom- and step-efficient manner using aqueous reaction conditions. Due to the structural diversity of oligonucleotide families, it is likely different biocatalytic strategies will be required for optimal production of different oligonucleotide structures.

**Figure 5:**
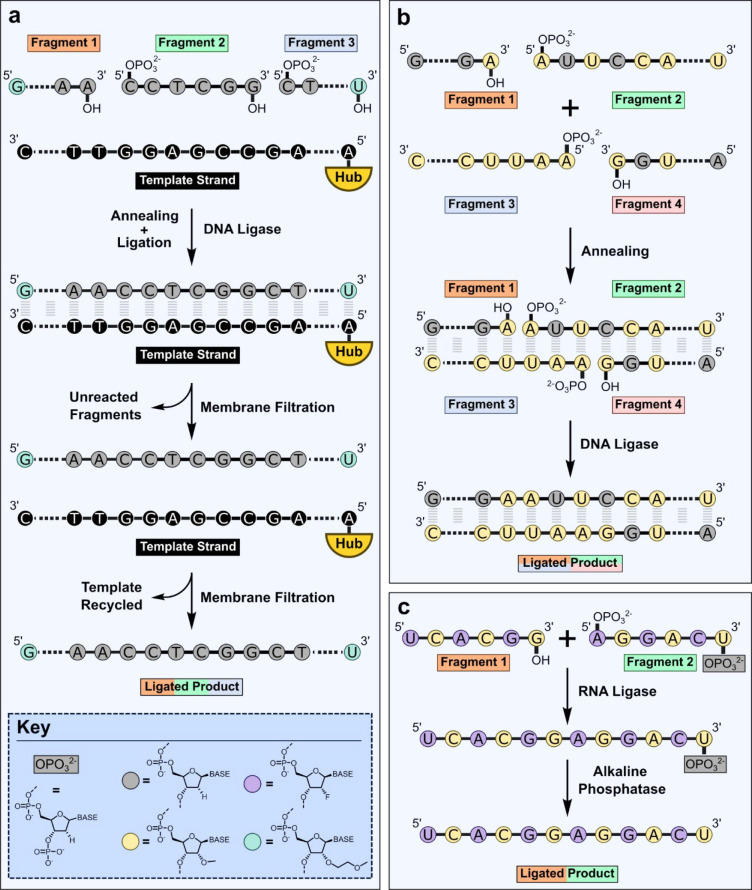
Convergent ligase catalyzed strategies for therapeutic oligonucleotide synthesis from short fragments. (a) Assembly of fragments annealed to a complementary template using DNA ligases. Impurity fragments with incorrect sequences do not anneal to the template and are removed by filtration. Following ligation, the oligonucleotide product is melted from the template and isolated by filtration, and the template is recycled. (b) Double-stranded oligonucleotides such as siRNAs can be constructed from overlapping, complementary fragments using a DNA ligase. (c) A nontemplated approach to oligonucleotide synthesis exploiting RNA ligases. 3′-Phosphate protecting groups are required to control the order of fragment assembly. Following ligation the 3′-end is dephosphoryated using alkaline phosphatase. The dashed lines between nucleotides indicate that some bases have been omitted from the diagram.

Ligase-catalyzed assembly of target oligonucleotides from short fragments (typically 3–8 nucleotides long) has proven a popular biocatalytic strategy (Figure 5).^71−74^ Shorter oligonucleotides are easier to produce with high purity using chemical methods, as byproducts such as truncated sequences arising from incomplete coupling reactions accumulate during each nucleotide addition. Furthermore, longer oligonucleotides can block solid supports leading to nonlinear flow rates that increases impurity levels. Longer sequences are also more challenging to resolve during chromatographic purification. Compared with linear chemical synthesis strategies, convergent biocatalytic approaches employing ligases should provide products in higher yield and overall purity and reduce the volume of acetonitrile required.^75,76^ Ligases use ATP or NAD to activate an oligonucleotide fragment containing a 5′-monophosphate group (fragment 1) and then couples the resulting adenylated sequence to the 3′-OH of a second oligonucleotide fragment.^77^ GSK have developed an approach using DNA ligases, which involves annealing multiple fragments to a complementary DNA template to control the order of assembly (Figure 5a).^74^ Using an engineered NAD-dependent ligase, three fragments containing 2′-*O*-methoxyethyl and phosphorothioate modifications were successfully ligated with 90% conversion. Impurity fragments containing base deletions, insertions, or mutations do not anneal efficiently to the template and can be removed by filtration through a membrane with an effective molecular weight cut off. The assembled product can then be melted from the template and isolated in a second filtration step. Three copies of the template are attached to a support “hub” molecule to increase the size of the template to allow facile separation from the product. Such templated approaches have the potential to generate oligonucleotides in high purity and avoid the need for costly chromatographic purification steps. Ajinomoto and Almac have also described an elegant DNA ligase-based approach for the synthesis of double-stranded siRNAs that does not require an external template. Here, both the sense and antisense strands are ligated from complementary overlapping fragments to deliver the target siRNA in a single step (Figure 5b).^72,73^ An alternative template-independent ligation strategy toward single stranded sequences involves the use of RNA ligases (RNAL).^73,78,79^ Here the donor 5′-monophosphorylated fragment (fragment 1) (Figure 5c) is synthesized with an additional 3′-phosphate protecting group to prevent uncontrolled polymerization. The 3′-phosphate can subsequently be removed following ligation using a phosphatase.^73^ Almac have developed a selectAZyme RNAL panel containing enzymes with activity toward a range of 2′-ribose modifications and phosphorothioate linkages, thus expanding the versatility of this approach.

**Figure 6:**
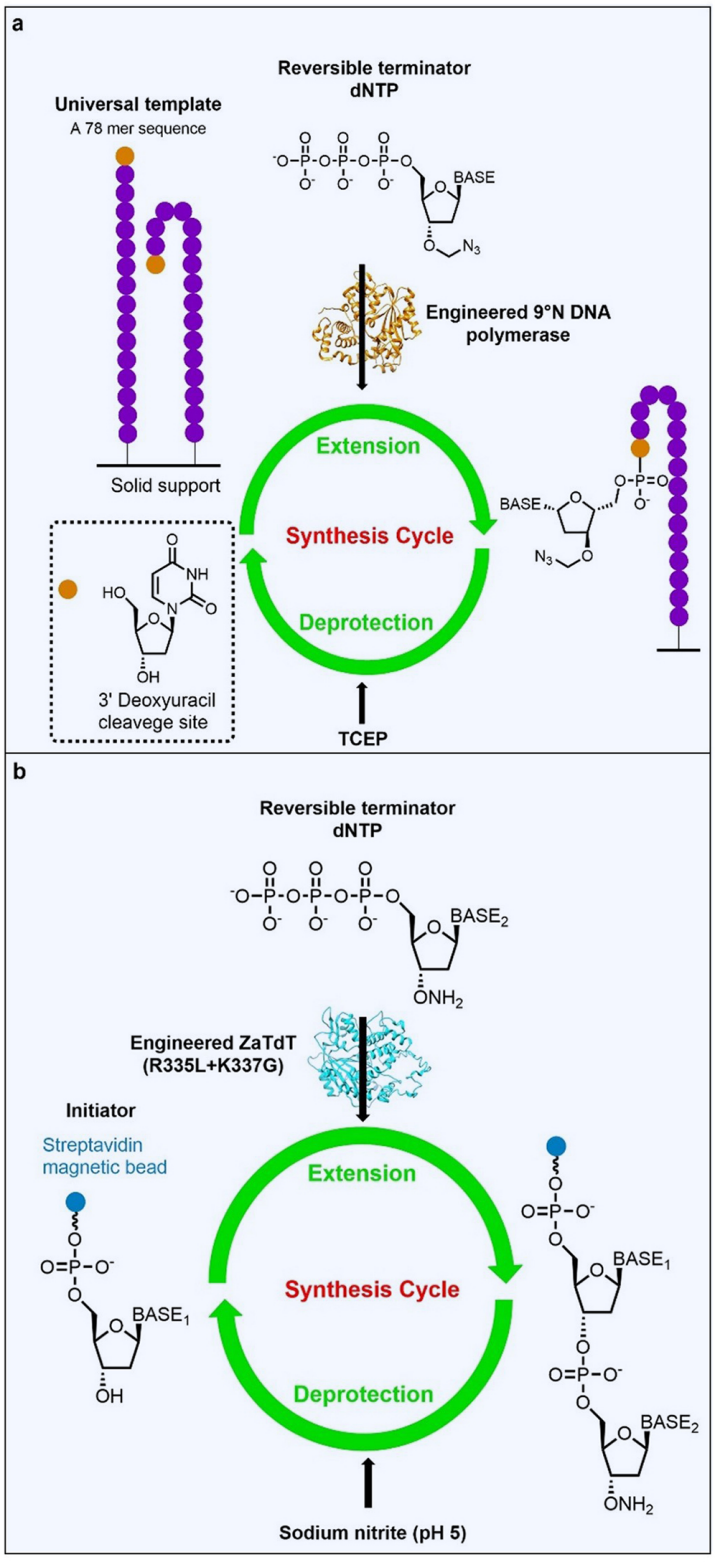
Oligonucleotide synthesis using DNA polymerases. (a) An approach exploiting an engineered 9°N polymerase and a universal template that contains all possible three base combinations with a 3′-deoxyuracil base. Following transient hybridization of the template to adjacent stands, the 3′-end is extended using 3′-*O*-azidomethyl-modified nucleotides. Following base addition, the reversible terminator is removed with TCEP to generate a free 3′-OH group which can undergo further extensions. The final oligonucleotide product is cleaved from the template at the deoxyuracil site using uracil-DNA glycosylase and apurinic/apyrimidic endonuclease. (b) A terminal deoxynucleotidyl transferase (TdT) approach for oligonucleotide synthesis. A R335L K337G variant of ZaTdT was used to extend an initiating sequence immobilized on steptavidin using 3′-ONH_2_-modified nucleotides. Following the extension step, the 3′-ONH_2_ blocking group is cleaved using sodium nitrite buffer.

Although not yet implemented on a large scale, the broad substrate promiscuity of wild-type ligases and the availability of engineered variants mean that ligation strategies have great potential to impact oligonucleotide manufacturing.^72−74^ However, the development of a sustainable biocatalytic platform for producing oligonucleotides will also require enzymatic approaches to fragment synthesis. Polymerase-based strategies for DNA synthesis have been investigated by several groups.^80−83^ DNA polymerases catalyze the extension of a priming sequence annealed to a template using nucleotide triphosphate building blocks. Hoff et al. designed a “universal template” (78 nucleotides long) that contains all possible three-base combinations (Figure 6a).^80^ Transient hybridization of the template to itself or an adjacent template molecule is sufficient for polymerase promoted addition of a 3′-protected nucleotide to the template 3′-end. The nucleobase sequence is controlled by sequential addition of the NTP building blocks which contain a 3′-*O*-azidomethyl reversible terminator. After nucleotide addition, excess NTP is removed and the 3′-OH group is deprotected using tris(2-carboxyethyl)phosphine (TCEP). Iterative cycles of coupling and deprotection provide the target sequence, which is cleaved from the template at a deoxyuracil site positioned at the 3′-end of the starting template, using a Uracil-DNA glycoylase in combination with an apurinic/apyrimidic endonuclease. A 9°N polymerase was selected, as variants with activity toward 3′-ribose substitutions have been previously reported.^84,85^ A variant with activity toward the 3′- blocking group was identified and used to produce a target 20mer oligonucleotide with an average coupling efficiency of 88%. Further purification of the substrate stocks, optimization, and automation is expected to substantially improve coupling efficiency to make this strategy suitable for larger scale synthesis.

A complementary approach involves the use of terminal deoxynucleotidyl transferases (TdTs), family X polymerases that promote template-independent oligonucleotide synthesis.^86^ Similar to the templated DNA polymerase strategy above, oligonucleotide sequences are generated through iterative rounds of coupling and deprotection steps using 3′-protected NTPs (Figure 6b).^87^ Engineered TdTs with activity toward 3′-reversible terminators have been developed for applications in DNA gene synthesis.^88−90^ For example, Lu et al. have developed a platform using a TdT from *Zonotrichia albicollis* (ZaTdT).^90^ R335L and K337G mutations were installed in the ZaTdT active site to accommodate a 3′-ONH_2_ protecting group, which can be cleaved following the coupling reaction using sodium nitrite buffer. A 98.7% average coupling efficiency was achieved across six extension cycles using the engineered ZaTdT. Although promising, several challenges need to be overcome to extend the utility of TdTs to therapeutic oligonucleotide synthesis. At present, large excesses of protected NTPs are used during coupling steps, which although suitable for small scale DNA synthesis are not practical for larger scale applications. Furthermore, available TdTs are poorly active toward 2′-ribose modifications and phosphorothioate linkages that are common in oligonucleotide therapies.^81,90^ RNALs have also been used for nontemplated oligonucleotide synthesis, where 5′-monophosphate (NMP) building blocks are coupled onto a growing oligonucleotide chain. Here, 3′-phosphate protecting groups are employed which can be removed enzymatically using an alkaline phosphatase.^73,74,91^ Compared with TdTs, RNALs are more tolerant of phosphorothioate linkages and 2′-ribose modifications including 2′-methoxy and 2′-fluoro motifs.^73,74^ However, at present RNALs still require an excess of ATP cosubstrate and NMP building blocks to achieve the high coupling efficiencies required for oligonucleotide synthesis.

## Outlook

Nucleosides are well-established as therapeutic agents for the treatment of viral infections and cancer and are also the central component of nucleotides, the building blocks required for CDN and oligonucleotide synthesis. Consequently, as the number of approved nucleic acid therapeutics gains momentum, cost-effective and streamlined synthetic routes to a wider range of noncanonical nucleosides are needed to underpin the field. Indeed, a recent summit of key industrial opinion leaders identified nucleoside synthesis as an emerging area of high potential impact.^92^ Several impressive biocatalytic cascades have been developed to synthesize nucleic acid therapeutics, which serve to illustrate the potential utility of enzymatic processes.^39,40,62^ During these endeavors, enzymes have been developed to tailor the nucleobase or to introduce 2′- and 4′-ribose modifications. To achieve more widespread utility moving forward, we must discover and engineer panels of biocatalysts that allow access to nucleosides containing a broader range of pharmaceutically relevant modifications.^93−95^ Developing routes to new nucleosides that are challenging to access with existing methods will also aid the discovery of new nucleic acid therapies with improved pharmacodynamic and pharmacokinetic properties.

To develop efficient routes to NTP and NMP building blocks required for CDN and oligonucleotide synthesis, enzymatic strategies to selectively elaborate nucleosides will prove invaluable. Strategies for selectively installing 5′-phosphate or 5′-triphopshate groups are already well established.^96−98^ Similarly, chemoenzymatic approaches to stereodefined thio-triphosphates have also been described.^62^ Less well developed are biocatalytic approaches to selectively modify the 3′-OH position of nucleosides and their derivatives. Such technology would greatly improve synthetic approaches to access the 3′-protected building blocks required for oligonucleotide fragment synthesis using polymerases, TdTs, or RNALs.^87,90,99^

Current synthetic routes to marketed oligonucleotides therapies do not attempt to control stereochemistry at phosphorothioate linkages, and consequently, drugs are administered to patients as complex mixtures of diastereoisomers, often with incomplete understanding of the biological effects of the individual isomers.^100−102^ Biocatalytic strategies could offer new opportunities to produce stereodefined oligonucleotide products for biological evaluation, potentially leading to the discovery of second-generation therapeutics with improved potency and patient safety. Indeed, wild-type polymerases, TdTs, and ligases are stereoselective and produce oligonucleotide products containing *R*_p_-linkages.^103−106^ A next key challenge is to engineer enantiocomplementary versions of these enzymes to allow access to a wide range of oligonucleotide stereoisomers.

In summary, the studies highlighted within this article demonstrate that biocatalysis has great potential to impact the synthesis of the entire portfolio of nucleic acid therapeutics. While challenges remain, with the combination of modern enzyme discovery tools and powerful enzyme engineering strategies, we are optimistic that biocatalysis will emerge as a key underpinning technology for the field.

## References

[ref1] Galmarini C. M.; Mackey J. R.; Dumontet C. Nucleoside Analogues and Nucleobases in Cancer Treatment. Lancet Oncol. 2002, 3, 415–424. 10.1016/S1470-2045(02)00788-X. 12142171

[ref2] De Clercq E. A Cutting-Edge View on the Current State of Antiviral Drug Development. Med. Res. Rev. 2013, 33, 1249–1277. 10.1002/med.21281. 23495004

[ref3] Jordheim L. P.; Durantel D.; Zoulim F.; Dumontet C. Advances in the Development of Nucleoside and Nucleotide Analogues for Cancer and Viral Diseases. Nat. Rev. Drug Discovery 2013, 12, 447–464. 10.1038/nrd4010. 23722347

[ref4] Shang M.; Lu K.; Guan W.; Cao S.; Ren M.; Zhou C. 2′,3′-Cyclic GMP-AMP Dinucleotides for STING-Mediated Immune Modulation: Principles, Immunotherapeutic Potential, and Synthesis. ChemMedChem. 2022, 17, e20210067 10.1002/cmdc.202100671. 34807508

[ref5] Danilchanka O.; Mekalanos J. J. Cyclic Dinucleotides and the Innate Immune Response. Cell 2013, 154, 962–970. 10.1016/j.cell.2013.08.014. 23993090 PMC3931520

[ref6] Bartsch T.; Becker M.; Rolf J.; Rosenthal K.; Lutz S. Biotechnological Production of Cyclic Dinucleotides-Challenges and Opportunities. Biotechnol. Bioeng. 2022, 119, 677–684. 10.1002/bit.28027. 34953086

[ref7] Khvorova A.; Watts J. K. The Chemical Evolution of Oligonucleotide Therapies of Clinical Utility. Nat. Biotechnol. 2017, 35, 238–248. 10.1038/nbt.3765. 28244990 PMC5517098

[ref8] Shen X.; Corey D. R. Chemistry, Mechanism and Clinical Status of Antisense Oligonucleotides and Duplex RNAs. Nucleic Acids Res. 2018, 46, 1584–1600. 10.1093/nar/gkx1239. 29240946 PMC5829639

[ref9] Kulkarni J. A.; Witzigmann D.; Thomson S. B.; Chen S.; Leavitt B. R.; Cullis P. R.; van der Meel R. The Current Landscape of Nucleic Acid Therapeutics. Nat. Nanotechnol. 2021, 16, 630–643. 10.1038/s41565-021-00898-0. 34059811

[ref10] Jiang M.; Chen P.; Wang L.; Li W.; Chen B.; Liu Y.; Wang H.; Zhao S.; Ye L.; He Y.; Zhou C. cGAS-STING, an Important Pathway in Cancer Immunotherapy. J. Hematol. Oncol. 2020, 13, 81 10.1186/s13045-020-00916-z. 32571374 PMC7310007

[ref11] Corrales L.; Hix Glickman L.; McWhirter S. M.; Kanne D. B.; Sivick K. E.; Katibah G. E.; Woo S.-R.; Lemmens E.; Banda T.; Leong J. J.; Metchette K.; Dubensky T. W. Jr.; Gajewski T. F. Direct Activation of STING in the Tumor Microenvironment Leads to Potent and Systemic Tumor Regression and Immunity. Cell Rep. 2015, 11, 1018–1030. 10.1016/j.celrep.2015.04.031. 25959818 PMC4440852

[ref12] Altman M. D.; Andresen B.; Chang W.; Childers M. L.; Cumming J. N.; Haidle A. M.; Henderson T. J.; Jewell J. P.; Liang R.; Lim J.; Liu H.; Lu M.; Nothrup A. B.; Otte R. D.; Siu T.; Trotter B. W.; Truong Q. T.; Walsh S. P.; Zhao K. Cyclic di-Nucleotide Compounds as STING Agonists. WO2017027646A1, 2016.

[ref13] Raal F. J.; Kallend D.; Ray K. K.; Turner T.; Koenig W.; Wright R. S.; Wijngaard P. L. J.; Curcio D.; Jaros M. J.; Leiter L. A.; Kastelein J. J. P. Inclisiran for Heterozygous Familial Hypercholesterolemia. N. Engl. J. Med. 2020, 382, 1520–1530. 10.1056/NEJMoa1913805. 32197277

[ref14] Lamb Y. N. Inclisiran: First Approval. Drugs 2021, 81, 389–395. 10.1007/s40265-021-01473-6. 33620677 PMC7900795

[ref15] Seley-Radtke K. L.; Yates M. K. The Evolution of Nucleoside Analogue Antivirals: A Review for Chemists and Non-Chemists. Part 1: Early Structural Modifications to the Nucleoside Scaffold. Antiviral Res. 2018, 154, 66–86. 10.1016/j.antiviral.2018.04.004. 29649496 PMC6396324

[ref16] McKenzie L. K.; El-Khoury R.; Thorpe J. D.; Damha M. J.; Hollenstein M. Recent Progress in Non-Native Nucleic Acid Modifications. Chem. Soc. Rev. 2021, 50, 5126–5164. 10.1039/D0CS01430C. 33644787

[ref17] Shelton J.; Lu X.; Hollenbaugh J. A.; Hyun Cho J.; Amblard F.; Schinazi R. F. Metabolism, Biochemical Actions, and Chemical Synthesis of Anticancer Nucleosides, Nucleotides, and Base Analogs. Chem. Rev. 2016, 116, 14379–14455. 10.1021/acs.chemrev.6b00209. 27960273 PMC7717319

[ref18] Li L.; Yin Q.; Kuss P.; Maliga Z.; Millán J. L.; Wu H.; Mitchison T. J. Hydrolysis of 2’3′-cGAMP by ENPP1 and Design of Nonhydrolyzable Analogs. Nat. Chem. Biol. 2014, 10, 1043–1048. 10.1038/nchembio.1661. 25344812 PMC4232468

[ref19] Eckstein F. Phosphorothioates, Essential Components of Therapeutic Oligonucleotides. Nucleic Acid Ther. 2014, 24, 374–387. 10.1089/nat.2014.0506. 25353652

[ref20] Eckstein F. Phosphorothioate Oligodeoxynucleotides: What Is Their Origin and What Is Unique About Them?. Antisense Nucleic Acid Drug Dev. 2000, 10, 117–121. 10.1089/oli.1.2000.10.117. 10805163

[ref21] Peifer M.; Berger R.; Shurtleff V. W.; Conrad J. C.; Macmillan D. W. A General and Enantioselective Approach to Pentoses: A Rapid Synthesis of PSI-6130, the Nucleoside Core of Sofosbuvir. J. Am. Chem. Soc. 2014, 136, 5900–5903. 10.1021/ja502205q. 24670208 PMC4210058

[ref22] Meanwell M.; Silverman S. M.; Lehmann J.; Adluri B.; Wang Y.; Cohen R.; Campeau L.-C.; Britton R. A Short *De Novo* Synthesis of Nucleoside Analogs. Science 2020, 369, 725–730. 10.1126/science.abb3231. 32764073

[ref23] Featherston A. L.; Kwon Y.; Pompeo M. M.; Engl O. D.; Leahy D. K.; Miller S. J. Catalytic Asymmetric and Stereodivergent Oligonucleotide Synthesis. Science 2021, 371, 702–707. 10.1126/science.abf4359. 33574208 PMC7961808

[ref24] Knouse K. W.; Degruyter J. N.; Schmidt M. A.; Zheng B.; Vantourout J. C.; Kingston C.; Mercer S. E.; Mcdonald I. M.; Olson R. E.; Zhu Y.; Hang C.; Zhu J.; Yuan C.; Wang Q.; Park P.; Eastgate M. D.; Baran P. S. Unlocking P(V): Reagents for Chiral Phosphorothioate Synthesis. Science 2018, 361, 1234–1238. 10.1126/science.aau3369. 30072577 PMC6349427

[ref25] Xu D.; Rivas-Bascón N.; Padial N. M.; Knouse K. W.; Zheng B.; Vantourout J. C.; Schmidt M. A.; Eastgate M. D.; Baran P. S. Enantiodivergent Formation of C-P Bonds: Synthesis of P-Chiral Phosphines and Methylphosphonate Oligonucleotides. J. Am. Chem. Soc. 2020, 142, 5785–5792. 10.1021/jacs.9b13898. 32109356

[ref26] Huang Y.; Knouse K. W.; Qiu S.; Hao W.; Padial N. M.; Vantourout J. C.; Zhen B.; Mercer S. E.; Lopez-Ogalla J.; Narayan R.; Olson R. E.; Blackmond D. G.; Eastgate M. D.; Schmidt M. A.; Mcdonald I. M.; Baran P. S. A P(V) Platform for Oligonucleotide Synthesis. Science 2021, 373, 1265–1270. 10.1126/science.abi9727. 34516793 PMC8579956

[ref27] Forbes K. C.; Jacobsen E. N. Enantioselective Hydrogen-Bond-Donor Catalysis to Access Diverse Stereogenic-at-P(V) Compounds. Science 2022, 376, 1230–1236. 10.1126/science.abp8488. 35679409 PMC9427129

[ref28] Kaspar E.; Stone M. R. L.; Neubauer P.; Kurreck A. A Route Efficiency Assessment and Review of the Synthesis of β-Nucleosides *via* N-Glycosylation of Nucleobases. Green Chem. 2021, 23, 37–50. 10.1039/D0GC02665D.

[ref29] Merino P. Chemical synthesis of nucleoside analogues; Wiley, 2013.

[ref30] Andrews B. I.; Antia F. D.; Brueggemeier S. B.; Diorazio L. J.; Koenig S. G.; Kopach M. E.; Lee H.; Olbrich M.; Watson A. L. Sustainability Challenges and Opportunities in Oligonucleotide Manufacturing. J. Org. Chem. 2021, 86, 49–61. 10.1021/acs.joc.0c02291. 33253568 PMC8154579

[ref31] Tedebark U.; Scozzari A.; Werbitzky O.; Capaldi D.; Holmberg L. Industrial-Scale Manufacturing of a Possible Oligonucleotide Cargo CPP-Based Drug. Methods Mol. Biol. 2011, 683, 505–524. 10.1007/978-1-60761-919-2_36. 21053153

[ref32] Molina A. G.; Sanghvi Y. S. Liquid-Phase Oligonucleotide Synthesis: Past, Present, and Future Predictions. Curr. Protoc. Nucleic Acid Chem. 2019, 77, e82 10.1002/cpnc.82. 30920171

[ref33] Bell E. L.; Finnigan W.; France S. P.; Green A. P.; Hayes M. A.; Hepworth L. J.; Lovelock S. L.; Niikura H.; Osuna S.; Romero E.; Ryan K. S.; Turner N. J.; Flitsch S. L. Biocatalysis. Nat. Rev. Methods 2021, 1, 46 10.1038/s43586-021-00044-z.

[ref34] Bornscheuer U. T.; Huisman G. W.; Kazlauskas R. J.; Lutz S.; Moore J. C.; Robins K. Engineering the Third Wave of Biocatalysis. Nature 2012, 485, 185–194. 10.1038/nature11117. 22575958

[ref35] Hauer B. Embracing Nature’s Catalysts: A Viewpoint on the Future of Biocatalysis. ACS Catal. 2020, 10, 8418–8427. 10.1021/acscatal.0c01708.

[ref36] Wu S.; Snajdrova R.; Moore J. C.; Baldenius K.; Bornscheuer U. T. Biocatalysis: Enzymatic Synthesis for Industrial Applications. Angew. Chem. Ind. Ed. 2021, 60, 88–119. 10.1002/anie.202006648. PMC7818486 32558088

[ref37] Adams J. P.; Brown M. J. B.; Diaz-Rodriguez A.; Lloyd R. C.; Roiban G.-D. Biocatalysis: A Pharma Perspective. Adv. Synth. Catal. 2019, 361, 2421–2432. 10.1002/adsc.201900424.

[ref38] Birmingham W. R.; Starbird C. A.; Panosian T. D.; Nannemann D. P.; Iverson T. M.; Bachmann B. O. Bioretrosynthetic Construction of a Didanosine Biosynthetic Pathway. Nat. Chem. Bio. 2014, 10, 392–399. 10.1038/nchembio.1494. 24657930 PMC4017637

[ref39] Huffman M. A.; Fryszkowska A.; Alvizo O.; Borra-Garske M.; Campos K. R.; Canada K. A.; Devine P. N.; Duan D.; Forstater J. H.; Grosser S. T.; Holst M. H.; Hughes G. J.; Jo J.; Joyce L. A.; Kolev J. N.; Liang J.; Maloney K. M.; Mann B. F.; Marshall N. M.; Mclaughlin M.; Moore J. C.; Murphy G. S.; Nawrat C. C.; Nazor J.; Novick S.; Patel N. R.; Rodriguez-Granillo A.; Robaire S. A.; Sherer E. C.; truppo M. D.; Whittaker A. M.; Verma D.; Xiao L.; Xu Y.; Yang H. Design of an *in vitro* Biocatalytic Cascade for the Manufacture of Islatravir. Science 2019, 366, 1255–1259. 10.1126/science.aay8484. 31806816

[ref40] McIntosh J. A.; Benkovics T.; Silverman S. M.; Huffman M. A.; Kong J.; Maligres P. E.; Itoh T.; Yang H.; Verma D.; Pan W.; Ho H.-I; Vroom J.; Knight A. M.; Hurtak J. A.; Klapars A.; Fryszkowska A.; Morris W. J.; Strotman N. A.; Murphy G. S.; Maloney K. M.; Fi P. S. Engineered Ribosyl-1-kinase Enables Concise Synthesis of Molnupiravir, an Antiviral for COVID-19. ACS Cent. Sci. 2021, 7, 1980–1985. 10.1021/acscentsci.1c00608. 34963891 PMC8704035

[ref41] Schrittwieser J. H.; Velikogne S.; Hall M.; Kroutil W. Artificial Biocatalytic Linear Cascades for Preparation of Organic Molecules. Chem. Rev. 2018, 118, 270–348. 10.1021/acs.chemrev.7b00033. 28481088

[ref42] Benítez-Mateos A. I.; Padrosa D. R.; Paradisi F. Multistep Enzyme Cascades as a Route Towards Green and Sustainable Pharmaceutical Syntheses. Nat. Chem. 2022, 14, 489–499. 10.1038/s41557-022-00931-2. 35513571

[ref43] Kaspar F.; Seeger M.; Westarp S.; Köllmann C.; Lehmann A. P.; Pausch P.; Kemper S.; Neubauer P.; Bange G.; Schallmey A.; Werz D. B.; Kurreck A. Diversification of 4’-Methylated Nucleosides by Nucleoside Phosphorylases. ACS Catal. 2021, 11, 10830–10835. 10.1021/acscatal.1c02589.

[ref44] Rabuffetti M.; Bavaro T.; Semproli R.; Cattaneo G.; Massone M.; Morelli C. F.; Speranza G.; Ubiali D. Synthesis of Ribavirin, Tecadenoson, and Cladribine by Enzymatic Transglycosylation. Catalysts 2019, 9, 355 10.3390/catal9040355.

[ref45] Benitez-Mateos A. I.; Paradisi F. Sustainable Flow-Synthesis of (Bulky) Nucleoside Drugs by a Novel and Highly Stable Nucleoside Phosphorylase Immobilized on Reusable Supports. ChemSusChem 2022, 15, e202102030 10.1002/cssc.202102030. 34726353 PMC9298701

[ref46] Nannemann D. P.; Kaufmann K. W.; Meiler J.; Bachmann B. O. Design and Directed Evolution of a Dideoxy Purine Nucleoside Phosphorylase. Protein Eng. Des. Sel. 2010, 23, 607–616. 10.1093/protein/gzq033. 20525731 PMC2898500

[ref47] Ohrui H.; Kohgo S.; Hayakawa H.; Kodama E.; Matsuoka M.; Nakata T.; Mitsuya H. 2′-Deoxy-4′-C-Ethynyl-2-Fluoroadenosine: A Nucleoside Reverse Transcriptase Inhibitor with Highly Potent Activity Against Wide Spectrum of HIV-1 Strains, Favorable Toxic Profiles, and Stability in Plasma. Nucleosides Nucleotides Nucleic Acids 2007, 26, 1543–1546. 10.1080/15257770701545218. 18066823

[ref48] Kaspar F.; Giessmann R. T.; Neubauer P.; Wagner A.; Gimpel M. Thermodynamic Reaction Control of Nucleoside Phosphorolysis. Adv. Synth. Catal. 2020, 362, 867–876. 10.1002/adsc.201901230.

[ref49] Rannes J. B.; Ioannou A.; Willies S. C.; Grogan G.; Behrens C.; Flitsch S. L.; Turner N. J. Glycoprotein Labeling Using Engineered Variants of Galactose Oxidase Obtained by Directed Evolution. J. Am. Chem. Soc. 2011, 133, 8436–8439. 10.1021/ja2018477. 21526835

[ref50] Syed Y. Y. Molnupiravir: First Approval. Drugs 2022, 82, 455–460. 10.1007/s40265-022-01684-5. 35184266 PMC8858220

[ref51] Painter G. R.; Bluemling G. R.; Natchus M. G.; Guthrie D. N4-Hydroxycytidine and Derivatives and Anti-Viral Uses Related Thereto. WO2019113462, 2018.

[ref52] Burke A. J.; Birmingham W. R.; Zhuo Y.; Thorpe T. W.; Zucoloto da Costa B.; Crawshaw R.; Rowles I.; Finnigan J. D.; Young C.; Holgate G. M.; Muldowney M. P.; Charnock S. J.; Lovelock S. L.; Turner N. J.; Green A. P. An Engineered Cytidine Deaminase for Biocatalytic Production of a Key Intermediate of the Covid-19 Antiviral Molnupiravir. J. Am. Chem. Soc. 2022, 144, 3761–3765. 10.1021/jacs.1c11048. 35224970 PMC8915250

[ref53] Sun L.; Wu J.; Du F.; Chen X.; Chem Z. J. Cyclic GMP-AMP synthase is a cytosolic DNA sensor that activates the type I interferon pathway. Science 2013, 339, 786–791. 10.1126/science.1232458. 23258413 PMC3863629

[ref54] Li X.; Shu C.; Yi G.; Chaton C. T.; Shelton C. L.; Diao J.; Zuo X.; Kao C. C.; Herr A. B.; Li P. Cyclic GMP-AMP Synthase is Activated by Double-Stranded DNA-Induced Oligomerization. Immunity 2013, 39, 1019–1031. 10.1016/j.immuni.2013.10.019. 24332030 PMC3886715

[ref55] Zhang X.; Wu J.; Du F.; Xu H.; Sun L.; Chen Z.; Brautigam C. A.; Zhang X.; Chen Z. J. The Cytosolic DNA Sensor cGAS Forms an Oligomeric Complex with DNA and Undergoes Switch-like Conformational Changes in the Activation Loop. Cell Rep. 2014, 6, 421–430. 10.1016/j.celrep.2014.01.003. 24462292 PMC3969844

[ref56] Burdette D. L.; Monroe K. M.; Sotelo-Troha K.; Iwig J. S.; Eckert B.; Hyodo M.; Hayakawa Y.; Vance R. E. STING is a direct innate immune sensor of cyclic di-GMP. Nature 2011, 478, 515–518. 10.1038/nature10429. 21947006 PMC3203314

[ref57] Wang H.; Hu S.; Chen X.; Shi H.; Chen C.; Sun L.; Chen Z. J. cGAS is essential for the antitumor effect of immune checkpoint blockade. Proc. Natl. Acad. Sci. U.S.A. 2017, 114, 1637–1642. 10.1073/pnas.1621363114. 28137885 PMC5320994

[ref58] Walsh C. T.; Tu B. P.; Tang Y. Eight kinetically stable but thermodynamically activated molecules that power cell metabolism. Chem. Rev. 2018, 118, 1460–1494. 10.1021/acs.chemrev.7b00510. 29272116 PMC5831524

[ref59] Chang W.; Altman M. D.; Lesburg C. A.; Perera S. A.; Piesvaux J. A.; Schroeder G. K.; Wyss D. F.; Cemerski S.; Chen Y.; DiNunzio E.; Haidle A. M.; Ho T.; Kariv I.; Knemeyer I.; Kopinja J. E.; Lacey B. M.; Laskey J.; Lim J.; Long B. J.; Ma Y.; Maddess M. L.; Pan B.-S.; Presland J. P.; Spooner E.; Steinhuebel D.; Truong Q.; Zhang Z.; Fu J.; Addona G. H.; Northrup A. B.; Parmee E.; Tata J. R.; Bennett D. J.; Cumming J. N.; Siu T.; Trotter B. W. Discovery of MK-1454: A Potent Cyclic Dinucleotide Stimulator of Interferon Genes Agonist for the Treatment of Cancer. J. Med. Chem. 2022, 65, 5675–5689. 10.1021/acs.jmedchem.1c02197. 35332774

[ref60] Yan H.; Wang X.; KuoLee R.; Chen W. Synthesis and Immunostimulatory Properties of the Phosphorothioate Analogues of cdiGMP. Bioorg. Med. Chem. Lett. 2008, 18, 5631–5634. 10.1016/j.bmcl.2008.08.088. 18799311

[ref61] Gaffney B. L.; Veliath E.; Zhao J.; Jones R. A. One-flask Syntheses of c-di-GMP and the [Rp,Rp] and [Rp,Sp] Thiophosphate Analogues. Org. Lett. 2010, 12, 3269–3271. 10.1021/ol101236b. 20572672 PMC2905038

[ref62] McIntosh J. A.; Liu Z.; Andresen B. M.; Marzijarani N. S.; Moore J. C.; Marshall N. M.; Borra-Garske M.; Obligacion J. V.; Fier P. S.; Peng F.; Forstater J. H.; Winston M. S.; An C.; Chang W.; Lim J.; Huffman M. A.; Miller S. P.; Tsay F.-R.; Altman M. D.; Lesburg C. A.; Steinhuebel D.; Trotter B. W.; Cumming J. N.; Northrup A.; Bu X.; Mann B. F.; Biba M.; Hiraga K.; Murphy G. S.; Kolev J. N.; Makarewicz A.; Pan W.; Farasat I.; Bade R. S.; Stone K.; Duan D.; Alvizo O.; Adpressa D.; Guetschow E.; Hoyt E.; Regalado E. L.; Castro S.; Rivera N.; Smith J. P.; Wang F.; Crespo A.; Verma D.; Axnanda S.; Dance Z. E. X.; Devine P. N.; Tschaen D.; Canada K. A.; Bulger P. G.; Sherry B. D.; Truppo M. D.; Ruck R. T.; Campeau L.-C.; Bennett D. J.; Humphrey G. R.; Campos K. R.; Maddess M. L. A Kinase-cGAS Cascade to Synthesize a Therapeutic STING Activator. Nature 2022, 603, 439–444. 10.1038/s41586-022-04422-9. 35296845

[ref63] Thillier V.; Sallamand C. C. B.; Vasseur J. J.; Debart F. Solid-Phase Synthesis of Oligonucleotide 5′-(α-P-Thio)triphosphates and 5′-(α-P-Thio)(β,γ-methylene)triphosphates. Eur. J. Org. Chem. 2015, 2015, 302–308. 10.1002/ejoc.201403381.

[ref64] Ludwig J.; Eckstein F. Rapid and Efficient Synthesis of Nucleoside 5′-O-(1-thiotriphosphates), 5′-Triphosphates, and 2’,3′-Cyclophosphorothioates using 2-Chloro-4H-1,3,2,-benzodioxaphosphorin-4-one. J. Org. Chem. 1989, 54, 631–635. 10.1021/jo00264a024.

[ref65] Rinaldi C.; Wood M. J. A. Antisense Oligonucleotides: The Next Frontier for Treatment of Neurological Disorders. Nat. Rev. Neurol. 2018, 14, 9–21. 10.1038/nrneurol.2017.148. 29192260

[ref66] Smith C. I. E.; Zain R. Therapeutic Oligonucleotides: State of the Art. Annu. Rev. Pharmacol. Toxicol. 2019, 59, 605–630. 10.1146/annurev-pharmtox-010818-021050. 30285540

[ref67] Dowdy S. F. Overcoming Cellular Barriers for RNA Therapeutics. Nat. Biotechnol. 2017, 35, 222–229. 10.1038/nbt.3802. 28244992

[ref68] Fire A.; Xu S.; Montgomery M. K.; Kostas S. A.; Driver S. E.; Mello C. C. Potent and Specific Genetic Interference by Double-Stranded RNA in. Caenorhabditis elegans. Nature 1998, 391, 806–811. 10.1038/35888. 9486653

[ref69] Beaucage S. L.; Caruthers M. H. Deoxynucleoside Phosphoramidites - A New Class of Key Intermediates for Deoxypolynucleotide Synthesis. Tetrahedron Lett. 1981, 22, 1859–1862. 10.1016/S0040-4039(01)90461-7.

[ref70] Knouse K. W.; Flood D. T.; Vantourout J. C.; Schmidt M. A.; Mcdonald I. M.; Eastgate M. D.; Baran P. S. Nature Chose Phosphates and Chemists Should Too: How Emerging P(V) Methods Can Augment Existing Strategies. ACS Cent. Sci. 2021, 7, 1473–1485. 10.1021/acscentsci.1c00487. 34584948 PMC8461637

[ref71] Kestemont D.; Herdewijn P.; Renders M. Enzymatic Synthesis of Backbone-Modified Oligonucleotides Using T4 DNA Ligase. Curr. Protoc. Chem. Biol. 2019, 11, e62 10.1002/cpch.62. 30688416

[ref72] Takahashi D.; Hagiwara Y.; Kajimoto S.; Konishi M. Method for Producing Modified Oligonucleotide Including Complementary Sequence. EP 3 929 205 A1, 2020.

[ref73] The Manufacturing Chemist Article, Towards the enzymatic synthesis of oligonucleotides, https://www.almacgroup.com/knowledge/wp-content/uploads/sites/10/2021/04/Man-Chem-Enzyme-synthesis-16.03.21-1.pdf (accessed 2022-08-01).

[ref74] Crameri A.; Tew D. G. Novel Processes for the Production of Oligonucleotides. WO2019121500A1, 2017.

[ref75] Zhou X.; Kiesman W. F.; Yan W.; Jiang H.; Antia F. D.; Yang J.; Fillon Y. A.; Xiao L.; Shi X. Development of Kilogram-Scale Convergent Liquid-Phase Synthesis of Oligonucleotides. J. Org. Chem. 2022, 87, 2087–2110. 10.1021/acs.joc.1c01756. 34807599

[ref76] Suchsland R.; Appel B.; Muller S. Synthesis of Trinucleotide Building Blocks in Solution and on Solid Phase. Curr. Protoc. Nucleic Acid Chem. 2018, 75, e60 10.1002/cpnc.60. 30375750

[ref77] Tomkinson A. E.; Vijayakumar S.; Pascal J. M.; Ellenberge T. DNA Ligases: Structure, Reaction Mechanism, and Function. Chem. Rev. 2006, 106, 687–699. 10.1021/cr040498d. 16464020

[ref78] Kikuchi Y.; Sakaguchi K. Enzymatic Synthesis of a Segment of Bacteriophage Qbeta Coat Protein Gene. Nucleic Acids Res. 1978, 5, 591–598. 10.1093/nar/5.2.591. 416426 PMC342004

[ref79] Uhlenbeck O. C.; Cameron V. Equimolar Addition of Oligoribonucleotides with T4 RNA Ligase. Nucleic Acids Res. 1977, 4, 85–98. 10.1093/nar/4.1.85. 17097 PMC342411

[ref80] Hoff K.; Halpain M.; Garbagnati G.; Edwards J. S.; Zhou W. Enzymatic Synthesis of Designer DNA Using Cyclic Reversible Termination and a Universal Template. ACS Synth. Biol. 2020, 9, 283–293. 10.1021/acssynbio.9b00315. 31895546

[ref81] Flamme M.; Hanlon S.; Iding H.; Puentener K.; Sladojevich F.; Hollenstein M. Towards the Enzymatic Synthesis of Phosphorothioate Containing LNA Oligonucleotides. Bioorg. Med. Chem. Lett. 2021, 48, 128242 10.1016/j.bmcl.2021.128242. 34217829

[ref82] Van Ness J.; Van Ness L. K.; Galas D. J. Isothermal Reactions for the Amplification of Oligonucleotides. Proc. Natl. Acad. Sci. U.S.A. 2003, 100, 4504–4509. 10.1073/pnas.0730811100. 12679520 PMC404692

[ref83] Chen T.; Romesberg F. E. A Method for the Exponential Synthesis of RNA: Introducing the Polymerase Chain Transcription (PCT) Reaction. Biochemistry 2017, 56, 5227–5228. 10.1021/acs.biochem.7b00846. 28930440

[ref84] Chen F.; Dong M.; Ge M.; Zhu L.; Ren L.; Liu G.; Mu R. The History and Advances of Reversible Terminators Used in New Generations of Sequencing Technology. Genom. Proteom. Bioinform. 2013, 11, 34–40. 10.1016/j.gpb.2013.01.003. PMC4357665 23414612

[ref85] Ju J.; Kim D. H.; Bi L.; Meng Q.; Bai X.; Li Z.; Li X.; Marma M. S.; Shi S.; Wu J.; Edwards J. R.; Romu A.; Turro N. J. Four-Color DNA Sequencing by Synthesis Using Cleavable Fluorescent Nucleotide Reversible Terminators. Proc. Natl. Acad. Sci. U.S.A. 2006, 103, 19635–19640. 10.1073/pnas.0609513103. 17170132 PMC1702316

[ref86] Bollum F. J. Thermal Conversion of Nonpriming Deoxyribonucleic Acid to Primer. J. Biol. Chem. 1959, 234, 2733–2734. 10.1016/S0021-9258(18)69770-4. 13802337

[ref87] Jensen M. A.; Davis R. W. Template-Independent Enzymatic Oligonucleotide Synthesis (TiEOS): Its History, Prospects, and Challenges. Biochemistry 2018, 57, 1821–1832. 10.1021/acs.biochem.7b00937. 29533604 PMC7995564

[ref88] Palluk S.; Arlow D. H.; de Rond T.; Barthel S.; Kang J. S.; Bector R.; Baghdassarian H. M.; Truong A. N.; Kim P. W.; Singh A. K.; Hillson N. J.; Keasling J. D. *De Novo* DNA Synthesis Using Polymerase-Nucleotide Conjugates. Nat. Biotechnol. 2018, 36, 645–650. 10.1038/nbt.4173. 29912208

[ref89] Eisenstein M. Enzymatic DNA Synthesis Enters New Phase. Nat. Biotechnol. 2020, 38, 1113–1115. 10.1038/s41587-020-0695-9. 33020638

[ref90] Lu X.; Li J.; Li C.; Lou Q.; Peng K.; Cai B.; Liu Y.; Yao Y.; Lu L.; Tian Z.; Ma H.; Wang W.; Cheng J.; Guo X.; Jiang H.; Ma Y. Enzymatic DNA Synthesis by Engineering Terminal Deoxynucleotidyl Transferase. ACS Catal. 2022, 12, 2988–2997. 10.1021/acscatal.1c04879.

[ref91] Schmitz C.; Reetz M. T. Solid-Phase Enzymatic Synthesis of Oligonucleotides. Org. Lett. 1999, 1, 1729–1731. 10.1021/ol990240n. 10836032

[ref92] Campos K. R.; Coleman P. J.; Alvarez J. C.; Dreher S. D.; Garbaccio R. M.; Terrett N. K.; Tillyer R. D.; Truppo M. D.; Parmee E. R. The Importance of Synthetic Chemistry in the Pharmaceutical Industry. Science 2019, 363, eaat0805 10.1126/science.aat0805. 30655413

[ref93] Arnold F. H. Directed Evolution: Bringing New Chemistry to Life. Angew. Chem., Int. Ed. 2018, 57, 4143–4148. 10.1002/anie.201708408. PMC5901037 29064156

[ref94] Turner N. J. Directed Evolution Drives the Next Generation of Biocatalysts. Nat. Chem. Biol. 2009, 5, 567–573. 10.1038/nchembio.203. 19620998

[ref95] Devine P. N.; Howard R. M.; Kumar R.; Thompson M. P.; Truppo M. D.; Turner N. J. Extending the Application of Biocatalysis to Meet the Challenges of Drug Development. Nat. Rev. Chem. 2018, 2, 409–421. 10.1038/s41570-018-0055-1.

[ref96] Fehlau M.; Kaspar F.; Hellendahl K. F.; Schollmeyer J.; Neubauer P.; Wagner A. Modular Enzymatic Cascade Synthesis of Nucleotides Using a (d)ATP Regeneration System. Front. Bioeng. Biotechnol. 2020, 8, 854 10.3389/fbioe.2020.00854. 32903716 PMC7438870

[ref97] Wu Y.; Fa M.; Tae E. L.; Schultz P. G.; Romesberg F. E. Enzymatic Phosphorylation of Unnatural Nucleosides. J. Am. Chem. Soc. 2002, 124, 14626–14630. 10.1021/ja028050m. 12465973

[ref98] Van Rompay A. R.; Johansson M.; Karlsson A. Phosphorylation of Nucleosides and Nucleoside Analogs by Mammalian Nucleoside Monophosphate Kinases. Pharmacol. Ther. 2000, 87, 189–219. 10.1016/S0163-7258(00)00048-6. 11008000

[ref99] Wojciechowski F.; Ybert T. Method for Preparing 3′-O-Amino-2’-Deoxyribonucleoside-5′-Triphosphates. US 20210300961, 2021.

[ref100] Iwamoto N.; Butler D. C. D.; Svrzikapa N.; Mohapatra S.; Zlatev I.; Sah D. W. Y.; Meena; Standley S. M.; Lu G.; Apponi L. H.; Frank-Kamenetsky M.; Zhang J. J.; Vargeese C.; Verdine G. L. Control of Phosphorothioate Stereochemistry Substantially Increases the Efficacy of Antisense Oligonucleotides. Nat. Biotechnol. 2017, 35, 845–851. 10.1038/nbt.3948. 28829437

[ref101] Koziolkiewicz M.; Krakowiak A.; Kwinkowski M.; Boczkowska M.; Stec W. J. Stereodifferentiation-The Effect of P Chirality of Oligo(Nucleoside Phosphorothioates) on the Activity of Bacterial RNase H. Nucleic Acids Res. 1995, 23, 5000–5005. 10.1093/nar/23.24.5000. 8559657 PMC307505

[ref102] Boczkowska M.; Guga p.; Stec W. J. Stereodefined Phosphorothioate Analogues of DNA: Relative Thermodynamic Stability of the Model PS-DNA/DNA and PS-DNA/RNA Complexes. Biochemistry 2002, 41, 12483–12487. 10.1021/bi026225z. 12369839

[ref103] Gupta A.; De Brosse C.; Benkovic S. J. Template-Prime-Dependent Turnover of (Sp)-dATP alpha S by T4 DNA Polymerase. The Stereochemistry of the Associated 3′ Goes to 5′-Exonuclease. J. Biol. Chem. 1982, 257, 7689–2692. 10.1016/S0021-9258(18)34436-3. 6282851

[ref104] Burgers P. M.; Eckstein F. A Study of the Mechanism of DNA Polymerase I from *Escherichia coli* with Diastereomeric Phosphorothioate Analogs of Deoxyadenosine Triphosphate. J. Biol. Chem. 1979, 254, 6889–6893. 10.1016/S0021-9258(18)50258-1. 378995

[ref105] Koziołkiewicz M.; Maciaszek A.; Stec W. J.; Semizarov D.; Victorova L.; Krayevsky A. Effect of P-Chirality of Oligo(Deoxyribonucleoside Phosphorothioate)s on the Activity of Terminal deoxyribonucleotidyl Transferase. FEBS Lett. 1998, 434, 77–82. 10.1016/S0014-5793(98)00900-4. 9738455

[ref106] Bryant F. R.; Benkovic S. J. Phosphorothioate Substrates for T4 RNA Ligase. Biochemistry 1982, 21, 5877–5885. 10.1021/bi00266a023. 7150532

